# Clinical and subclinical maternal hypothyroidism and their effects on neurodevelopment, behavior and cognition

**DOI:** 10.20945/2359-3997000000201

**Published:** 2020-03-04

**Authors:** Alice Batistuzzo, Miriam Oliveira Ribeiro

**Affiliations:** 1 Departamento de Pós-Graduação em Distúrbios do Desenvolvimento Centro de Ciências Biológicas e da Saúde Universidade Presbiteriana Mackenzie São Paulo SP Brasil Departamento de Pós-Graduação em Distúrbios do Desenvolvimento, Centro de Ciências Biológicas e da Saúde (CCBS), Universidade Presbiteriana Mackenzie (UPM), São Paulo, SP, Brasil

**Keywords:** Hypothyroidism, pregnancy, offspring, cognition, behavior

## Abstract

Clinical and subclinical hypothyroidism are the most common hormonal dysfunctions during pregnancy. Insufficient maternal thyroid hormones (THs) in the early stages of pregnancy can lead to severe impairments in the development of the central nervous system because THs are critical to central nervous system development. In the fetus and after birth, THs participate in neurogenic processes, cell differentiation, neuronal activation, axonal growth, dendritic arborization, synaptogenesis and myelination. Although treatment is simple and effective, approximately 30% of pregnant women in Brazil with access to prenatal care have their first consultation after the first trimester of pregnancy, and any delay in diagnosis and resulting treatment delay may lead to cognitive impairment in children. This review summarizes the effects of clinical and subclinical hypothyroidism on fetal neurodevelopment, behavior and cognition in humans and rodents. Arch Endocrinol Metab. 2020;64(1):89-95

## INTRODUCTION

Thyroid hormones (THs) are essential for the development, growth and metabolism of all vertebrates from the embryonic period onward. Thyroxine, or T4 (3,5,3’,5’-tetraiodothyronine), the principal product of the thyroid gland, is considered a prohormone from which triiodothyronine (T3) is derived by deiodination. T3 is an active TH capable of binding to high affinity receptors located in the nucleus of its target cells, the thyroid receptors (TRs). T3 is a biologically active hormone and is fundamental for growth, differentiation, activity regulation and organ and tissue metabolism, even in adult life ( 1 ).

The fetal thyroid becomes fully functional in humans from the second trimester of gestation. However, the fetus expresses TRs for THs from the 9th week ( 2 ), suggesting that THs are important even before the fetus is able to synthesize them. The source of THs during the first trimester is, therefore, exclusively maternal, and changes in TH availability may cause complications during pregnancy and have a negative effect on neurodevelopment, including consequences for behavior and cognition ( 3 ). The more severe the change, the greater the damage ( 4 ). However, controversy still exists regarding whether subclinical hypothyroidism (SCH) impacts the neurodevelopment and cognition of offspring.

## METHODS

The review process took place between January and December 2018. The PubMed, Lilacs, Scopus and Web of Science databases were used with no restriction on the date of publication. A bibliographic search was conducted using the following keywords in English, Portuguese and Spanish: hypothyroidism, subclinical hypothyroidism, gestational, pregnancy, offspring, cognition, behavior and memory. Papers related to congenital hypothyroidism were excluded from the analysis.

## LITERATURE REVIEW

### The fetal thyroid and the role of THs in central nervous system development during pregnancy

THs are essential for the development, growth and metabolism of all vertebrates from the embryonic stage to adulthood. The thyroid gland, through its interaction with the thyroid stimulating hormone (TSH), which binds with membrane receptors present in the thyroid follicular cell, secretes two hormones: T4 and T3 ( 5 ).

Two pathways guarantee the production of adequate T3 levels: thyroid activity regulated by the hypothalamic-pituitary-thyroid system and peripheral T3 generation from T4, which depends on the action of specific enzymes called iodothyronine deiodinases. About 80% of circulating T3 is generated from T4 deiodination, whereas 20% is generated by the thyroid itself ( 6 ).

Deiodinase type 2 (D2) converts T4 to T3. It is highly expressed in the central nervous system (CNS), the pituitary gland, brown adipose tissue and the placenta and provides T3 for the tissues in which it is expressed. Deiodinase type 3 (D3) is primarily responsible for the degradation of THs through the conversion of T4 into reverse T3 (rT3) and the conversion of T3 into T2. In adults, it is found mainly in the CNS and in the skin, and its action is increased in hyperthyroidism. However, it is highly expressed in fetal tissues ( 7 ), the placenta ( 8 ), the gravid uterus and umbilical vessels ( 9 ), ensuring that the fetus is not overexposed to active T3 ( 10 ).

**Figure 1:**
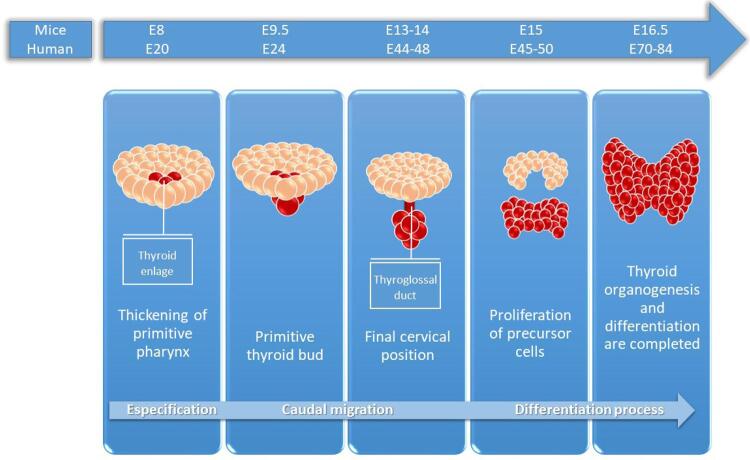
Main phases of thyroid development from the 8th day of embryonic development (E8) for mice and the 20th day of embryonic development (E20) for humans.

The thyroid is the first endocrine gland to be formed during embryonic development ( 11 ). Its organogenesis can be described in 3 phases ( Figure 1 ).

**Phase 1** . This phase begins on the 20th day of embryonic development (E20) in humans and on the 8th day (E8) in mice, when endodermal thickening is observed on the floor of the pharynx, giving rise to a set of cells called the thyroid primordium. The thyroid primordium invaginates, forming the primitive thyroid bud, which begins its caudal migration. The coding genes for *TTF-1* , *TTF-2* and *PAX-8* are expressed at the beginning of glandular formation and are involved in the migration and proliferation process, but they are not dependent on T3 signaling ( 12 , 13 ).

**Phase 2** . At the 7th week (E44-48) in humans and at the end of the 2nd week (E13-14) in mice, the thyroid bud reaches the final cervical position anterior to the trachea. At the end of the migration, the differentiation process depends on the progressive activation of thyroglobulin ( *TG* ), thyroid peroxidase ( *TPO* ), *TSH* receptor ( *TSHR* ), sodium/iodine symporter ( *NIS* ) and pendrin ( *PDS* ), all of which are related to TH biosynthesis ( 11 ). From the 9th week (E63) in humans and E11.5 in mice, the expression of the TRs ( 5 ) is already detectable. The TRs belong to a superfamily of hormone-responsive nuclear transcription factors and interact with specific sequences in target genes called thyroid hormone-responsive elements (TREs) ( 14 ). Interaction between the receptors and the responsive elements can regulate positive and negative gene expression. In the case of promoters containing positive TREs, T3 must bind to the TRs to stimulate gene expression that is usually suppressed in the absence of such binding ( 15 ). Conversely, the activity of promoters containing negative TREs is stimulated when the receptors are not bound to T3, and transcription is suppressed if T3 binds to the TRs ( 14 , 15 ).

**Phase 3.** During the period comprising the 10th (E70) and 12th weeks (E84) in humans and the third week in mice (E16.5), thyroid organogenesis and differentiation are completed. Between the 12th and 14th weeks of fetal development, the human thyroid begins producing THs. This increases progressively until reaching its maximum capacity at week 28. In mice, the thyroid becomes active from E.16.5 ( 11 , 16 ).

This sequence of events strongly suggests that maternal THs are critical to proper fetal development. The TH transporters organic anion-transporting polypeptide 1c1 ( *Oatp1c1* ) and monocarboxylate transporter 8 ( *MCT8* ) are present in the placenta and the blood–brain barrier, which ensures their availability to the fetus ( 16 - 18 ).

**Figure 2:**
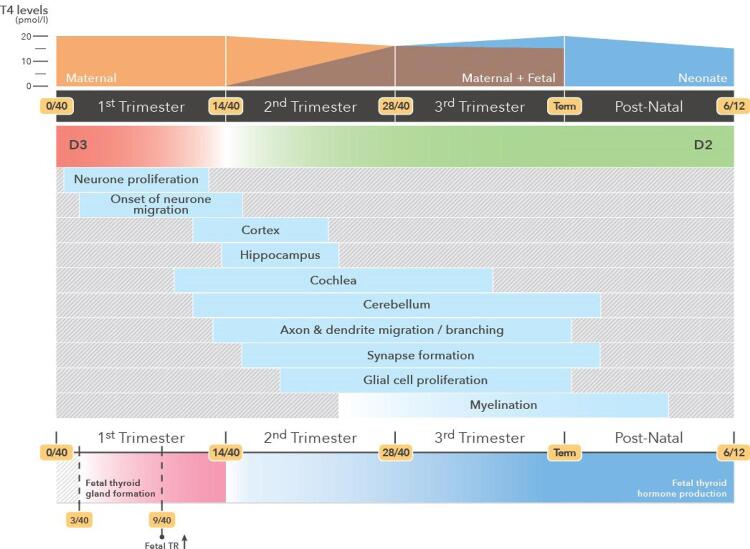
Development of fetal thyroid, expression of receptors, expression of deiodinases 2 and 3, levels of maternal and fetal T4 and CNS development throughout the gestational period in humans (adapted from [16]).

From the 9th week of gestation, T3 of maternal origin regulates the neuronal proliferation processes and the initiation of neuronal migration in the cerebral cortex, hippocampus and medial ganglionic eminence in the fetus ( Figure 2 ) ( 16 , 19 ). From the 14th week, the fetus starts to contribute progressively to TH supply. At this stage, neurogenesis, neuronal migration, axonal growth, dendritic arborization, synaptogenesis, glial cell differentiation and myelination onset occur ( 16 ).

From the 28th week, the mother and fetus contribute equally to available TH levels and to the ongoing maturation of the CNS. However, despite increasing fetal TH levels, the thyroid is not fully mature until birth, and insufficient maternal TH levels may still cause adverse effects ( 12 , 16 , 19 ). After birth, the child depends exclusively on the synthesis of THs by its thyroid, and the process of myelination, the migration of granular cells in the dentate gyrus of the hippocampus and cerebellum, pyramidal cells in the cortex and Purkinje cells in the cerebellum continues ( 16 ).

It is reported in the literature that thyroid disorders during pregnancy are associated with severe maternal, fetal and neonatal complications such as miscarriage ( 20 ), preterm delivery ( 21 ) and preeclampsia ( 22 ).

### Maternal hypothyroidism

During gestation, the demand for TH production by the maternal thyroid increases significantly, by approximately 20% to 50%, to maintain a euthyroid state. During the first week of gestation, the placenta begins synthesizing human chorionic gonadotropin (hCG), which acts as a TSH receptor agonist. Maximum hCG concentration is reached between the 9th and 11th weeks, after which it decreases again, and a stable concentration is maintained from the 20th week. Simultaneously, there is an increase in thyroxine-binding globulin (TBG) levels and the action of type 3 deiodinase in the placenta. Together, these mechanisms result in increased maternal T4 serum concentration during the first trimester and a relative decrease over the second and third trimesters ( 23 , 24 ). Thus, conditions that affect the adequate availability of T4 may affect both the mother and the fetus ( 25 ).

Clinical hypothyroidism (CH) is defined as a situation in which subnormal free T4 levels is accompanied by TSH levels higher than 10 mIU/L ( 26 ), and its incidence ranges from 0.3 to 0.5% in pregnant women ( 27 ). Subclinical hypothyroidism (SCH) is defined elevated TSH (> 4.5mIU/L) accompanied by normal free T4 levels ( 27 ). Its prevalence varies according to population, region, age, sex and race. Studies with large populations in various countries have reported a prevalence of 3 to 17% in the adult population, with higher frequencies in women and elderly people ( 25 , 28 ). It has a high prevalence among pregnant women of between 2 and 4% ( 29 ).

In pregnant women, the most common cause of CH and SCH is autoimmune thyroiditis ( 30 ). In this situation, the increase in TSH is accompanied by an increase in anti-peroxidase (anti-TPO) and anti-thyroglobulin (anti-TBG) antibodies ( 26 , 28 ). Other causes include iodine deficiency, thyroid destruction (iodine ablation or surgery), and – more rarely –hypothalamic-pituitary disorders ( 31 ).

### Changes in neurodevelopment and cognition in humans due to hypothyroidism

Maternal CH is associated with a number of adverse effects in the mother and child. Decreased THs in the fetus, especially during the first trimester of pregnancy, lead to fetal damage that includes impaired nerve cell differentiation, inadequate CNS development, increased risk of perinatal defects, low birth weight, and motor and cognitive developmental impacts ( 15 , 25 ).

Both maternal CH and SCH can cause IQ reduction in the child ( 32 ). Vulsma and cols. ( 33 ) compared the development of 120 children born to mothers diagnosed with hypothyroidism at the 12th week of gestation during the first 2 years of life and found impairments in the children’s motor and intellectual development compared to children born to euthyroid mothers. Korevaar and cols. ( 34 ) studied nearly 4,000 child–mother pairs and reported that the children of mothers with hypo- and hyperthyroxinemia during pregnancy had significantly lower IQ scores than the children of mothers with adequate free T4. This decrease in IQ – assessed at 6 years old – was accompanied by a reduction in gray matter and the volume of the cortex. Ghassabian and cols. ( 35 ) also observed a reduction in verbal and nonverbal cognitive development among children of mothers with hypothyroxinemia, but without changes in brain morphology.

SCH’s impact on pregnant woman is better established than its impact on the fetus, and considerable controversy surrounds its influence on offspring cognition ( 36 ). It is associated with higher rates of placental abruption and increased risk of preterm birth ( 37 ), spontaneous abortion ( 38 ), gestational hypertension and severe preeclampsia ( 39 ).

Preterm birth is the most common cause of neuropsychological dysfunction in children and is associated with reduced IQ and attention deficit hyperactivity disorder ( 40 , 41 ). Behavioral disorders such as aggression and emotional disorders such as depression and anxiety are often associated with low gestational age ( 41 ).

Therefore, SCH, by increasing the chances of preterm birth, may increase the chances of possible cognitive impairment, even if indirectly.

However, there is no consensus on whether treatment with levothyroxine (L-T4) efficiently prevents cognitive impairments. Haddow and cols. ( 32 ) suggested that increasing T4 doses during pregnancy for hypothyroid women may be sufficient to ensure the delivery of T3 and prevent the cognition impairments associated with low T4. However, Casey and cols. ( 42 ) found no better cognitive outcomes when comparing the offspring of treated and untreated women with SCH or hypothyroxinemia.

### Changes in neurodevelopment, gene expression and cognition in rodents

Several studies involving rats and mice have demonstrated how maternal hypothyroidism influences cognitive function in offspring. Opazo and cols. ( 43 ) reported impairments in operational memory, spatial learning and changes in the expression and distribution of proteins involved in the formation of synapses in the offspring of hypothyroid rats, such as the discs large MAGUK scaffold protein 4 ( *Dlgh-4* ) and glutamate ionotropic receptor NMDA type subunit 1 ( *Grin1* ).

Brain-derived neurotrophic factor ( *BNDF* ) is an important protein involved in neurogenesis, neuronal maintenance and synaptic plasticity in the hippocampus, cortex and brain stem. It also promotes long-term potentiation (LTP) in the hippocampus ( 44 ). *BDNF* reduction in the hippocampus was correlated with impairment in spatial learning and operational memory in males and females born to mothers with CH ( 45 - 47 ). This correlation is also found in the offspring of both sexes born to mothers with SCH ( 48 ).

Migration and differentiation processes are dependent on reelin, a glycoprotein involved in the signaling of neuronal migration in the cortex, hippocampus and cerebellum, and upregulated by the action of T3 ( 49 ). Both clinical and subclinical maternal hypothyroidism result in a decrease in reelin levels and aberrant neuronal migration in rats ( 46 , 50 ). The impairment in learning and spatial memory can be reversed in rodents with L-T4 replacement during the initial period of fetal development, around embryonic day 13. However, if the replacement is performed later, the impairments are not reversed.

In addition, the gene that encodes the Sonic Hedgehog ( *SHH* ) signaling protein is positively regulated by T3 and plays a key role in embryonic development. The *SHH* signaling protein is involved in neurogenesis, the localization of dopaminergic and serotoninergic neurons, and the mediation of neuroprotective and neurotrophic effects in several types of neurons ( 49 ). In rats, maternal hypothyroidism results in reduced *Shh* expression and changes in cerebellar morphology ( 51 - 53 ).

Several other proteins regulated by T3 are involved in memory and learning: *EGR-1 is* a transcription factor that increases significantly during the *LTP* process; the activity-regulated cytoskeletal protein ( *ARC* ) is involved with post-learning neuronal plasticity through the dendritic system; and Ras-proximate-1 protein ( *RAP-1* ) participates in the processes of proliferation and neuronal survival, adhesion and differentiation ( 54 ). Mitogen-activated protein kinases ( *MAPK* ) also constitute a family of important neurotransmitters and growth factor-activated markers, such as the MAPK activating protein ( *MEK* ) and the extracellular signal regulated kinase ( *ERK* ) ( 54 ).

In rats with CH and SCH, the expression of *Egr-1* , *Arc* , *Erk* and *Bndf* in their offspring’s hippocampus was significantly decreased, and *RAP-1* levels were increased when compared to the offspring of euthyroid rats, although this was more pronounced in the HC roup. Changes in these proteins’ expression are accompanied by impairments in spatial learning evaluated using the water maze test and exploratory behavior evaluated using the open field test. These effects probably occur because these proteins are part of the *Ras/Raf/MEK/ERK* signaling pathway, which is involved in the LTP process ( 54 , 55 ).

In another study, a contradictory finding was observed in which increased *Erk 1/2* protein levels were found in the hippocampus of the offspring of rats with SCH. The authors argued that this discrepancy in the data, in which *Erk* levels appears to sometimes increase and sometimes decrease, was caused different experimental designs, such as the hippocampal region analyzed, the time elapsed since the thyroidectomy, whether the thyroidectomy was partial or total and the analyses performed ( 55 ).

The consequences of gestational CH for both mother and offspring are widely recognized, and hormone replacement therapy with L-T4 is recommended. However, there is still some debate regarding the treatment of pregnant women diagnosed with SCH ( 42 ). Some authors argue that the treatment of pregnant women with SCH is not recommended due to lack of evidence and randomized clinical trials on the benefits of levothyroxine replacement for both mother and fetus ( 37 , 56 , 57 ).

## CONCLUSION

In conclusion, the consequences for CNS development and cognition caused by insufficient maternal THs in CH are well known in humans and have been established experimentally in rodents. However, rodent studies indicate that SCH also impacts cognitive development.

Since 2000, the Brazilian Ministry of Health has recommended a minimum of 6 prenatal consultations, according to the *Programa de Humanização no Pré-natal e Nascimento* (PHPN), the Program for the Humanization of Prenatal Care and Childbirth ( 58 ). However, the 2018 Firjan Municipal Development Index (FDI) reported that, in 2016, one-third of pregnant women did not receive the recommended minimum number of prenatal consultations ( 59 ). In 2014, an epidemiological study conducted in Brazil indicated that, among pregnant women who received prenatal care, 24.2% started it after the 16^th^ gestation week ( 60 ). Therefore, monitoring thyroid status throughout pregnancy, especially during the first trimester, is essential to ensuring adequate fetal development. Although prenatal TH testing should be routine to allow for the timely identification and treatment of any changes, not all pregnant women have adequate access to exams. More studies are necessary to evaluate whether SCH impacts cognition and behavior in humans, as studies in animal models mimicking this pathology have shown damage to the fetus.
